# A genome‐wide analysis of the USDA Soybean Isoline Collection

**DOI:** 10.1002/tpg2.20310

**Published:** 2023-03-29

**Authors:** Erin Gilbert, Ryan Merry, Benjamin W. Campbell, Robert M. Stupar, Aaron J. Lorenz

**Affiliations:** ^1^ Department of Agronomy and Plant Genetics University of Minnesota Saint Paul MN USA

## Abstract

The USDA Soybean Isoline Collection has been an invaluable resource for the soybean genetics and breeding community. This collection, established in 1972, consists of 611 near‐isogenic lines (NILs) carrying one or multiple genes conferring traits that had been determined to exhibit Mendelian inheritance. It has been used in multiple studies on the genetic basis, physiology, and agronomy of these qualitative traits. Here, we used publicly available genotype (SoySNP50K), phenotype, and pedigree data on this collection to characterize the isogenicity of the NILs and identify chromosomal positions of unmapped genes. A total of 368 NILs had at least 80% identity to their recurrent parent and, thus, were useful for what can be called introgression mapping. Both on‐target and off‐target introgressions were evaluated. The size of on‐target introgressions into individual NILs ranged from 61 kb to 8.4 Mb, whereas off‐target introgressions ranged from 2.6 kb to 54.8 Mb. The observed large off‐target introgressions indicated that some NILs carry introgressions nearly the size of an entire chromosome. By applying introgression mapping to genes that had never been mapped, we identified the likely chromosomal positions of six such genes: *ab*, *im*, *lo*, *Np*, *pc*, and *Rpm*. The size of mapping intervals was large in some cases (10.28 Mb for *im*) but small in others (0.21 Mb for *Np*). The results reported herein will provide future researchers with a resource to help select informative NILs for future studies, and provide a starting point to further fine map, and ultimately clone and functionally characterize these six soybean genes.

## Core Ideas

- A total of 368 NILs had at least 80% identity to their recurrent parent and thus were useful introgression mapping.
- The size of on‐target introgressions into individual NILs ranged from 61 kb to 8.4 Mb, whereas off‐target introgressions ranged from 2.6 kb to 54.8 Mb.
- Chromosomal positions of six genes (*ab, im, lo, Np, pc*, and *Rpm*) were mapped for the first time.

## 1 INTRODUCTION

Numerous qualitative traits in soybean have been characterized by geneticists and breeders since the beginning of scientific studies on soybean cultivation (Owen, 1927a, 1927b, 1928; Piper & Morse, 1910; Woodworth, 1932). Because of the world‐wide economic importance of this legume crop, many of these soybean traits have been, and continue to be, of practical relevance, such as disease resistance (e.g., race‐specific resistance to Phytophthora root and stem rot) (Bernard et al., 1957; Dorrance et al., 2016) and growth habit (e.g., stem termination type) (Thompson et al., 1997; Woodworth, 1932). The study of some traits has contributed to a better understanding of the agronomic value of variants in soybean morphology (Baldocchi et al., 1983; Hartung et al., 1980, 1981; Specht et al., 1985) and physiology (e.g., chlorophyll deficiency) (Chen & Palmer, 1998). Still others have been useful to breeders as phenotypic markers (e.g., flower and pubescence color) (Bernard, 1975; Woodworth, 1932). Many of these traits have been compiled into the so‐named USDA “T” (for “Type”) collection from outside sources (Nelson, 2011; Palmer et al., 2004). This collection has been an invaluable starting point for a wide range of studies on soybean genetics, physiology, and breeding (reviewed by Palmer et al., 2004).

Near‐isogenic lines (NILs), which can be created through multiple rounds of backcrossing (Kaeppler et al., 1993) or derived from heterogenous inbred families (Tuinstra et al., 1997), are useful for studying the effects of genes in a common genetic background. They have frequently been used in studies on the genetics of soybean traits, including photoperiod response (e.g., Cober et al., 1996) abiotic stress tolerance (e.g., Atencio et al., 2021; Merry et al., 2019), soybean aphid resistance (e.g., Ajayi‐Oyetunde et al., 2016), Phytophthora root rot resistance (e.g., Lin et al., 2014), and stem termination type (Thompson et al., 1997), among many other traits. The USDA Soybean Isoline Collection is a collection of NILs created to help facilitate studies on the effect of a specific gene in a common genetic background (Bernard et al., 1991). The USDA Soybean Isoline collection contains over 100 nuclear alleles representing 81 unique traits. The genes and traits in this collection have been used in genetic studies and assessing potential for commercial use (Bernard et al., 1991). Many of these genes originated from the lines in the USDA Soybean Genetic Type Collection (Palmer et al., 2004) which were introgressed into the backgrounds of cultivars Clark, Harosoy, Williams, or another appropriate cultivar (Bernard et al., 1991). This introgression was often achieved using backcrossing schemes that typically involved five backcrossing generations, although a consistent scheme was not used among all traits and families. In some cases, NILs of the same genetic background were crossed together to combine traits (Bernard et al., 1991).

The development of this NIL collection stretched over two decades, with the first release in 1972, the second in 1975 (coinciding with the first Soybean Genetics Newsletter publication), and the last in 1991 (Bernard et al., 1991). Since its release, the USDA Soybean Isoline collection has been referenced in over 100 publications, including scientific articles, textbooks, and newsletters covering topics such as maturity (Watanabe et al., 2009), disease resistance (Yu et al., 1996), yield (Guzman et al., 2007), and genomic structure (Stec et al., 2013). Muehlbauer et al. (1991) studied a subset of NILs in the Isoline collection to associate Restriction Fragment Length Polymorphism (RFLP) markers to the qualitative traits, placing four loci on an early version of a soybean RFLP map.

Though this collection has been available for almost half a century, we aimed to develop a single easily accessible reference which summarizes the collection in terms of pedigree, traits isolated, the extent to which the NILs are isogenic, and the status of gene discovery and validation. Such a resource would be valuable for future researchers using this collection. Moreover, the majority of the NILs comprising this collection have been genotyped with the SoySNP50K array by Song et al. (2015). The NIL genotype data can be used to map many traits not previously placed on a genetic or physical map, and possibly refine map positions of traits only roughly mapped to date. In light of this, the objectives of this study were to combine historical phenotypic data collected by Bernard et al. (1991) and modern genotyping data from the SoySNP50K BeadChip (Song et al., 2015) to first characterize the collection as an isoline resource for soybean researchers, and second to use introgression mapping as a means of chromosomal positioning of the donor parent‐contributed genomic segments containing the genes that govern the traits in this soybean NIL collection.

**Core Ideas**

- A total of 368 NILs had at least 80% identity to their recurrent parent and thus were useful for introgression mapping.
- The size of on‐target introgressions into individual NILs ranged from 61 kb to 8.4 Mb, whereas off‐target introgressions ranged from 2.6 kb to 54.8 Mb.
- Chromosomal positions of six genes (*ab, im, lo, Np, pc*, and *Rpm*) were mapped for the first time.

## 2 MATERIALS AND METHODS

### 2.1 Germplasm and genotyping

Pedigree information on the 611 NILs in the USDA Soybean Isoline Collection was acquired from Dr. Randy Nelson (formerly USDA‐ARS). Those pedigrees were cross‐checked against the original collection pedigree information provided in Bernard et al. (1991). Although Bernard generated most of the NILs via a standard 5‐backcross introgression of a donor parent (DP) trait into a recurrent parent (RP), it is important to note that multiple crossing schemes were often used to generate the multi‐trait class of NILs. This included, in many cases, combining traits from different DP sources into a single NIL by crossing two BC5‐derived NILs, each carrying just one trait. In other cases, multiple DPs were introduced in different BC (backcross) generations of NIL development. These non‐traditional BC mating schemes complicated the genomic analysis of these multi‐trait NILs, but introgression mapping was still possible in many cases, so those NILs were not entirely eliminated from the current study. For downstream analyses, we applied genetic and genomic approaches consistent with BC5‐derived NILs, but the variation among NIL pedigrees was also considered in our data interpretation.

**FIGURE 1:**
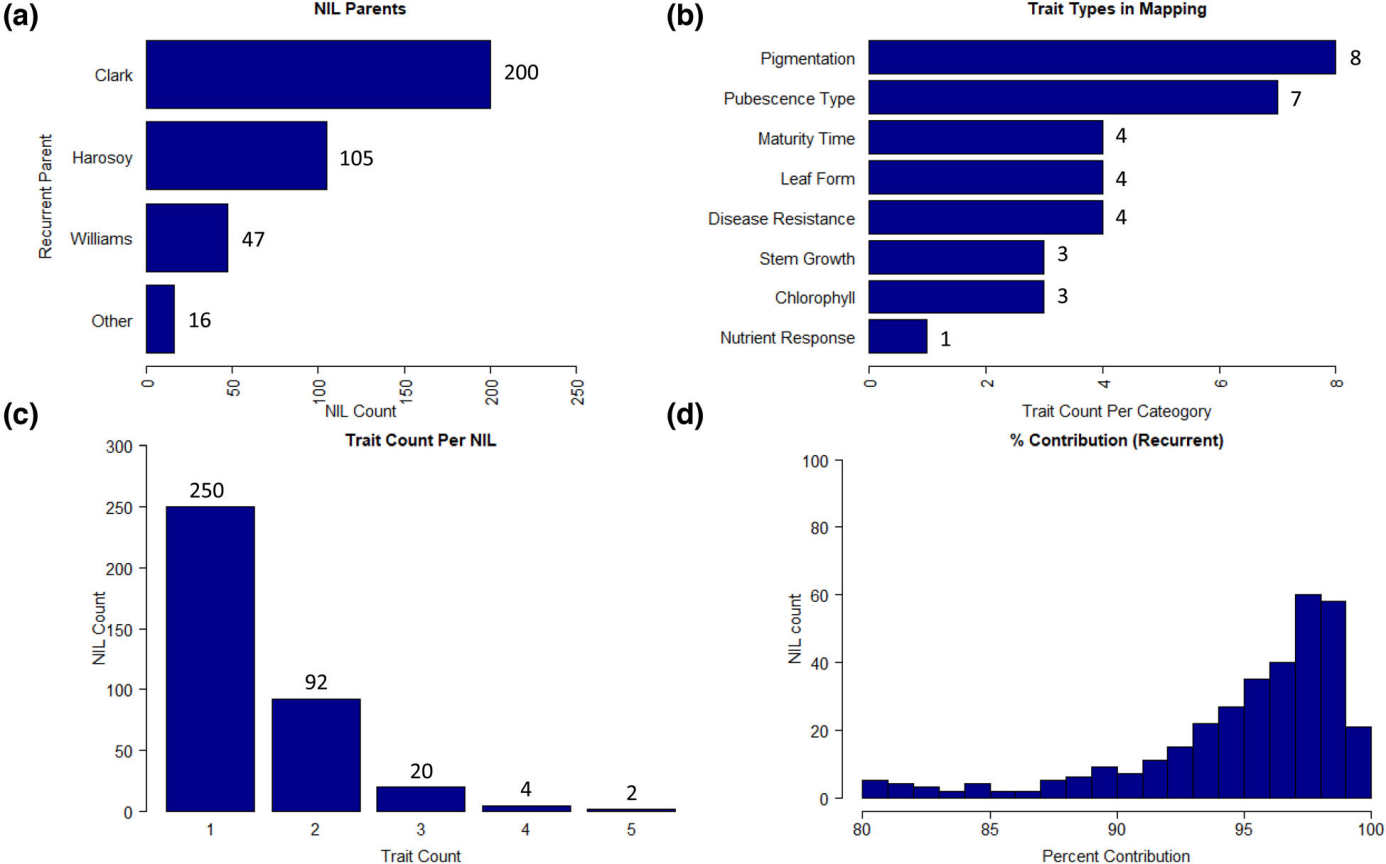
Summary of the mapping population used in this study. (a) The distribution of recurrent parents used by Bernard to develop the mapping near‐isogenic lines (NILs). (b) The categories which the traits mapped in this study fall into. (c) The distribution of the number of mapped traits isolated in each NIL. (d) The distribution of recurrent parent contribution for the NILs used in this mapping study.

Genotypic data on the NILs collected using the SoySNP50K BeadChip were made available as part of the genotyping of the entire USDA Soybean Germplasm Collection by Song et al. (2015), which is publicly available at Soybase (https://soybase.org/snps/). Not all 611 NILs in the original collection could be used in this study for introgression mapping. This method of chromosomal positioning of the gene‐containing introgressed segment can be done only if a given NIL meets the following criteria (1) the trait in the NIL did not arise from a spontaneous mutation of nuclear or cytoplasmic gene in the RP; (2) SoySNP50K genotype data had to be available for the NIL and its RP; and (3) the genomic contribution of the RP to the NIL had to be at least 80% (based on a marker similarity analysis described below). Criterion #1 removed 48 NILs, criterion #2 removed 123 NILs, and criterion #3 removed 72 NILs. The remaining 368 NILs contained 34 introgressed traits that could be mapped. This NIL set represented three major RPs (Clark, Harosoy, and Williams) and 16 minor RPs (Figure 1a), plus 75 unique DPs that were confirmed by tracing the pedigrees of each NIL back to the original cross. In many cases, the donor listed in the NIL pedigree was a previously developed NIL, but the pedigrees of the latter NILs were then used to trace back to the original donor line. Single nucleotide polymorphism (SNP) genotype data was not available for the DP in 18 of the 350 NILs, but could be imputed based on the detection of non‐RP SNP marker segments present in those 18 NILs that were clearly not inherited from the RP; these imputed intervals were assumed to be DP introgressions and treated as such in the analysis. This imputation is valid under the condition that the RP is homozygous and homogeneous, which is a valid assumption for the USDA Soybean Germplasm Collection as this collection is a pure line collection and thus, highly homozygous and homogeneous (Mihelich et al., 2020; Nelson, 2011). Observed heterozygosity for the SoySNP50K data was observed to be less than 0.1% for the recurrent parents used in this study.

### 2.2 Isogenicity of the NILs within the collection

We estimated the degree of genetic identity between each NIL and its respective RP, a value we termed isogenicity. For NILs with genotype data available on both parents (both the DP and RP), the isogenicity between the RP and NIL was estimated as follows (Bernardo, 2020):

(1) $$\mathrm{Contribution}\;\mathrm{of}\;\mathrm{RP}\;\mathrm{to}\;\mathrm{NIL}\;=\;\frac{S_{\mathrm{RP}/\mathrm{NIL}}\;-\;\left(S_{\mathrm{RP}/\mathrm{DP}}\;\times \;S_{\mathrm{DP}/\mathrm{NIL}}\right)}{1\;-\;{\left(S_{\mathrm{RP}/\mathrm{NIL}}\right)}^{2}}$$

where $S_{\mathrm{RP}/\mathrm{DP}}$ is the marker identity between the RP and DP, $S_{\mathrm{RP}/\mathrm{NIL}}$ is the marker identity between the RP and NIL, and $S_{\mathrm{DP}/\mathrm{NIL}}$ is the marker identity between the DP and NIL. NILs for which multiple traits were donated from multiple donor parents were analyzed individually with each DP and then the sum of these donor contributions was subtracted from one to estimate the RP contribution. This calculation assumes the SoySNP50K markers are uniformly distributed, which is valid for the genetic map (Song et al., 2016), but not necessarily for the physical map (Song et al., 2013). Therefore, these estimates of isogenicity should be considered as approximations based on marker similarity and the genetic space, not precise estimates of physical genome similarity. Calculations were made with custom scripts using the base package of R (v4.0.2; R Core Team, 2021).

### 2.3 Introgression analysis

Physical positions on the Wm82.a2.v1 map for the SoySNP50K, SNPs were obtained from Soybase (https://soybase.org/snps/). To analyze the introgressions based on recombination frequency, we approximated the genetic map positions for these SNPs using the ‘Williams82' × PI479752 genetic map published by Song et al. (2016). The positions of the SNPs that were not included on this genetic map because they were not polymorphic in the Williams82 × PI479752 cross were approximated via interpolation executed with LOESS regression using the *loess* function with default settings in R (R Core Team, 2021) that related the marker genetic position with the Wm82.a2.v1 physical position.

Introgressed DP genomic segments in a NIL were delineated based on the presence of homozygous SNP marker alleles that were identifiable as originating from the DP. The DP‐contributed SNP alleles separated by 2.58 cM or less were combined to define a single DP haplotype introgression. The 2.58 cM threshold was based on an enumeration of the number of possible events that could lead to an effective double crossover between SNP markers at the BC5 generation. The events included a double crossover in the BC1 generation, a single crossover in the BC1 combined with a single crossover in the BC2, a single crossover in the BC1 and single crossover in the BC3, and so forth. This amounted to 15 possible events that could lead to an effective double crossover (effDC), for which the probability is *P*(effDC) = 15*c* ^2^, where *c* is the recombination frequency. No crossover interference was assumed, as the majority of the second crossover events in our model would have occurred in a different generation than the first crossover, and thus represent independent meiosis events. We set *P*(effDC) = 0.01 and solved for *c*, that is, we solved for the recombination frequency between markers that would lead to a 1 − *P*(effDC) = 0.99 probability, an effective double recombination event was not observed between markers, and therefore a sequence of DP alleles could be assumed to form a single haplotype introgression. We used Morgan's map function (cM = *c* × 100) to relate recombination frequency to cM based on the linear relationship between *c* and cM at small distances (Penalba & Wolf, 2020). The size of the introgression was defined as the physical length between the start and stop SNP. The R script used to execute this analysis is provided in the Supporting Information 1.

### 2.4 Introgressed trait mapping

Mapping of each trait (Table 1) was carried out by combining SoySNP50K BeadChip data with pedigree and trait information on the Collection (Bernard et al., 1991). The number of polymorphic SNPs between DP and RP pairs ranged from 179 to 19,227, with a median of 14,301. To start, NILs sharing a common trait were pooled and heterozygous SNPs were removed. SNPs were re‐coded as either matching the DP SNP (0) or the RP SNP (1) at all polymorphic loci. Introgressed regions (scores of 0) were compared across NILs by comparing the proportion of scores across NILs sharing a trait. This step in the mapping process involved calculating the probability that each donor SNP was subjected to selection versus being inherited by chance alone. To do this, we used the binomial probability function as follows:

(2) $$P\;=\;\left(\begin{array}{c} n\\ x \end{array}\right)\;\theta^{x}\;{\left(1\;-\;\theta \right)}^{n\;-\;x}$$

where, for any given SNP, *n* is the total number of pooled NILs carrying the trait in question (i.e., total number in pool), *x* is the number of NILs that inherited the DP SNP, and *θ* is the probability the NIL inherited the DP allele by chance alone. Theta was approximated as 0.0156, which corresponds to the expected genetic contribution of the donor parent to BC5‐derived NILs (Fehr, 1991). This approximation was used because the vast majority of NILs were derived from the BC5 generation. It ignores the fact that some NILs may have been derived from the same BC1, BC2, etc. family. This was a necessary simplifying assumption we need to make to enable our use of historical data lacking complete pedigree records.

**TABLE 1:** List of 33 genes evaluated using introgression mapping in the USDA Soybean Isoline Collection (Bernard et al., 1991). The near‐isogenic lines were created via backcross‐based introgression of the genes contributed by various donor parents into the recurrent parents listed for each gene.

Gene	Trait description	Trait category	Recurrent parents
*ab*	Delayed leaf abscission	Leaf form	Clark, Harosoy
*d1*	Nuclear “stay green,” chlorophyll presence after senescence	Chlorophyll	Clark, Harosoy
*d2*	Nuclear “stay green,” chlorophyll presence after senescence	Chlorophyll	Clark, Harosoy
*dt1*	Determinate stem	Stem growth	Clark, Harosoy, Williams, Other
*Dt2*	Semi‐determinate stem	Stem growth	Clark, Harosoy, Williams
*E1*	Late maturity	Maturity time	Clark, Harosoy
*e2*	Early maturity	Maturity time	Clark, Harosoy, Williams, Other
*e3*	Early maturity, low photoperiod sensitivity to incandescent light	Maturity time	Clark, Harosoy, Other
*G*	Green seed coat	Chlorophyll	Clark, Harosoy
*im*	Non‐mottling under SMV infection	Pigmentation	Clark, Williams
*L1*	Black pod	Pigmentation	Clark, Harosoy
*l2*	Tan pod	Pigmentation	Clark, Harosoy
*Lf1*	Five‐foliate leaf	Leaf form	Clark, Harosoy
*ln*	Narrow leaflet	Leaf form	Clark, Harosoy
*lo*	Oval leaflet, few‐seeded pod	Leaf form	Clark, Harosoy
*Np*	Phosphorous tolerant	Nutrient response	Clark, Harosoy
*P1*	Glabrous (Pubescence density)	Pubescence	Clark, Harosoy
*p2*	Puberulent	Pubescence	Clark, Harosoy
*pa1*	Semi‐appressed pubescence (upper leaf surface)	Pubescence	Clark, Harosoy
*pa2*	Appressed pubescence with *pa1*	Pubescence	Clark, Harosoy
*pc*	Curly pubescence	Pubescence	Clark, Harosoy
*Pd1*	Dense pubescence	Pubescence	Clark, Harosoy
*Ps*	Sparse pubescence	Pubescence	Clark, Harosoy
*rmd*	Susceptible to powdery mildew	Disease resistance	Clark, Williams
*Rpm*	Resistance to downy mildew	Disease resistance	Clark, Williams, Other
*Rsv2*	Resistant to soybean mosaic potyvirus infection	Disease resistance	Williams
*rxp*	Resistant to bacterial pustule	Disease resistance	Clark, Harosoy, Other
*S*	Short internode length	Stem growth	Clark, Harosoy
*t*	Pubescence color	Pigmentation	Clark, Harosoy
*td*	Light tawny to near‐grey pubescence with *T*	Pigmentation	Clark
*w1*	White flower	Pigmentation	Clark, Harosoy, Williams
*w4*	Near‐white flower	Pigmentation	Clark, Harosoy
*wm*	Magenta flower	Pigmentation	Clark, Harosoy

The logarithm to the base 10 of the probabilities obtained from equation (2) were multiplied by −1 and plotted to visualize the results in Manhattan‐style plots. To first identify SNPs that may have been selected (i.e., introgressed with the trait gene) during NIL development, a significance threshold of −log(*P*) = 4 was used, representing a p≤0.0001 that the DP SNP was inherited by chance alone.

To delineate a genomic interval from these significant SNPs, we used a simple algorithm that identified the SNP locus with the greatest −log_10_(*P*) value and arbitrarily designated the genomic location of this SNP as the center of the introgressed interval. Interval boundaries were determined based on the closest upstream and downstream RP scores in any given NIL, which constituted the narrowest interval. Visual inspection of coded allelic scores was performed to confirm fully introgressed genomic regions surrounding the target gene in all NILs carrying the trait and distinguish likely target regions from spurious associations, mis‐called SNPs, and mis‐mapped SNPs. Validation of our introgression mapping results for the various NIL trait genes relative to previously mapped intervals of identical genes was accomplished via a literature search. These successes validated our methods as a proof of concept and increased our confidence in the newly mapped intervals in this study. Calculations and algorithms were performed with custom scripts using the base package of R (v4.0.2; R Core Team, 2021). The scripts used for the introgression mapping as described above are provided in the Supporting Information 1.

## 3 RESULTS

### 3.1 Population characterization

The NILs in the USDA Soybean Isoline Collection were developed primarily by backcrossing qualitative traits into the background of three public soybean cultivars—Clark (maturity Group IV), Harosoy (maturity group II), and Williams (maturity group III)—widely grown during the 1970s and 1980s (Hartwig, 1973; Mikel et al., 2010). Of the 368 NILs we selected from the full collection of 611 for genetic analysis, based on the criteria described in the Materials and Methods section, 200 were in a Clark background (PI548533, PI547464, PI547406), 105 were in a Harosoy background (PI548573, PI547679, PI548221, PI548575, PI548631), 47 were in a Williams background (PI548631, PI518672), and 16 were in some other RP background, such as the cultivars Chippewa or Corsoy (Figure 1a). Notably, the donor parent in these 16 NILs was either Clark, Harosoy, or Williams (Bernard et al., 1991). PI548533 is the plant introduction number for Clark, whereas PI547464 ('L64‐2244') and PI547406 ('L61‐5448') are Clark isolines that each have greater than 99% marker similarity to Clark according the SoySNP50K data. PI548573 is Harosoy, whereas PI547679 ('L61‐5047'), PI548221 ('T239'), and PI548575 ('Harosoy 63') are Harosoy isolines that have 99.4%, 95.3%, and 99.0% marker similarity to Harosoy, respectively. PI548631 is Williams, whereas PI518671 is Williams82 with 96.7% marker similarity to Williams.

Note that 34 DP‐source alleles had been introgressed into these 368 NILs (Figure 1b), with many of these alleles introduced into more than one RP and often into multiple NILs of each RP. However, most of the 368 NILs harbored just one gene (250) or just two genes (92) governing the target traits (Figure 1c). Bernard generated 20, 4, and 2 NILs in which he respectively pyramided three, four, and five genes (Figure 1c). Among the retained NILs the isogenicity between each NIL and its RP was greater than 85% in the vast majority of cases, with the mean and median being 95.7% and 96.8%, respectively (Figure 1d). The RP marker similarity of each NIL in this subset is provided in Table S1.

**FIGURE 2:**
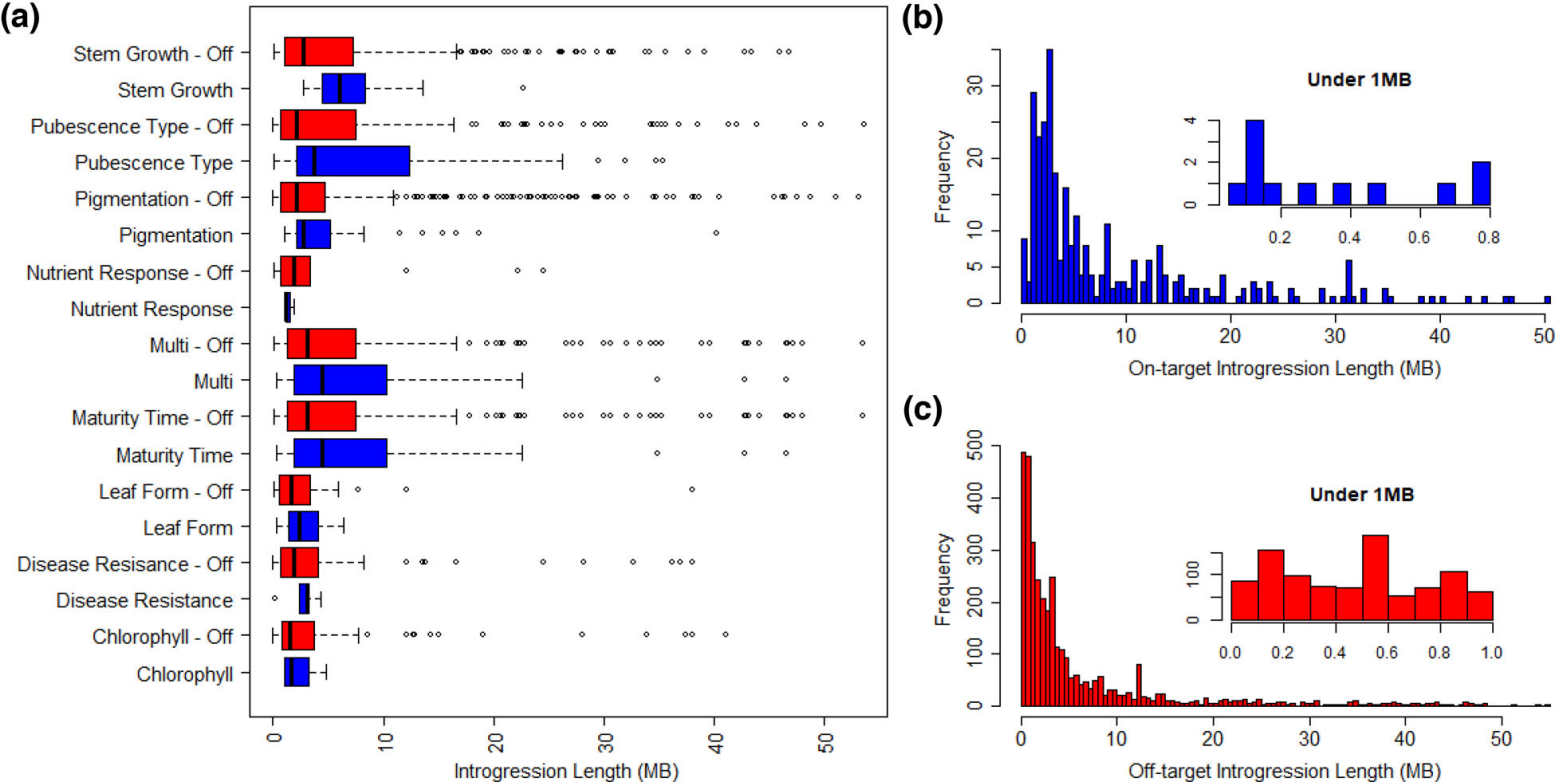
Near‐isogenic lines (NILs) used in mapping had many on‐ and off‐target donor regions introgressed into their genomes. (a) The distribution of the length of donor parent introgressions for both off‐ (red) and on‐target (blue) introgressions grouped by trait type. (b) Overall size distribution of all on‐target introgression among all NILs used for mapping in this study. (c) Overall size distribution of all off‐target introgression among all NILs used for mapping in this study.

A total of 3924 independent introgressions were captured among the 368 NILs. Of those introgressions, 498 were on‐target, meaning they overlapped with the predicted intervals for the traits isolated in their respective NILs, whereas 3426 were off‐target (Figure 2). The mean number of off‐target introgressions per NIL was 9.2 with a median of 6. The average size of the off‐target introgressions was 6.4 Mb, while it was 8.4 Mb for on‐target introgressions. The range in length of off‐target introgressions was 2.6 kb to 54.8 Mb, while it was 61 kb to 50.1 Mb for on‐target introgressions (Figure 2). Introgression segments, defined as described in the Materials and Methods, along with introgressed individual SNPs across the 20 nuclear chromosomes for each NIL are displayed in images provided in the Supporting Information 2.

### 3.2 Introgression mapping results

The entire collection originally described by Bernard et al. (1991) included 69 independent genes each controlling a simply inherited trait. The traits were categorized into 10 types by Bernard et al. (1991); chlorophyll, disease resistance, leaf form, maturity time, nutrient response, pigmentation, pubescence type, stem growth, seed composition, and other (which included cytoplasmic genes). In this study, introgession mapping was applied to the 368 NILs possessing 42 of the 69 trait genes. Of those 42 genetic loci, 33 (i.e., 79%) genes spanning eight trait categories were successfully mapped (Table 1; Figure 1b). Of the 33 mapped loci, 20 had been cloned in previous studies (Table 2), seven had been mapped previously but not yet cloned (Table 3), and six were mapped for the first time in this study (Table 4).

**TABLE 2:** Trait genes mapped using the USDA Soybean Isoline Collection that have been cloned in previous studies. The mapped intervals coordinates are in reference to the Wm82.a2.v1 genome assembly, as is the cloned gene location.

	Results from Isoline Collection mapping				Cloned genes as reported in previous studies		
Trait	Mapped interval (chr:bp)	Interval size (Mb)	Number of NILs^a^	Overlap^b^	Study	Gene model	Gene location (chr:bp)
*d1*	Gm01:54014967‐55079646	1.06	19	Yes	Fang et al. (2014)	Glyma.01g214600	Gm01:54554210‐54556460
*d2*	Gm11:1630295‐2630521	1.00	19	Yes	Fang et al. (2014)	Glyma.11g027400	Gm11:1974063‐1976315
*G*	Gm01:53455419‐54444658	0.99	11	No	Wang et al. (2018).	Glyma.01g198500	Gm01:53226421‐53229986
*Ln*	Gm20:35645532‐36177520	0.53	23	Yes	Jeong et al. (2012)	Glyma.20g116200	Gm20:35827672‐35830107
*E1*	Gm06:19447386‐20353073	0.91	37	Yes	Xia et al. (2012)	Glyma.06g207800	Gm06:20207077‐20207940
*e2*	Gm10:45226484‐45546527	0.32	54	Yes	Watanabe et al. (2011)	Glyma.10g221500	Gm10:45294735‐45316121
*e3*	Gm19:47238127‐47709441	0.47	16	Yes	Watanabe et al. (2009)	Glyma.19g224200	Gm19:47633059‐47641958
*L1*	Gm19:35677408‐36987863	1.31	6	No	He et al. (2015).	Glyma.19g101700	Gm19:34914719‐34919684
*T*	Gm06:17761410‐18970072	1.21	57	Yes	Toda et al. (2002)	Glyma.06g202300	Gm06:18731105‐18738025
*w1*	Gm13:17307263‐18644670	1.34	20	Yes	Zabala and Vodkin (2007)	Glyma.13G072100	Gm13:17312472‐17317198
*w4*	Gm17:39154316‐39521449	0.37	5	No	Yan et al. (2014)	Glyma.17g252200	Gm17:40652090‐40657097
*Wm*	Gm13:17272186‐20701079	3.43	3	Yes	Takahashi et al. (2007)	Glyma.13g082300	Gm13:19154278‐19159357
*p2*	Gm20:1582950‐2148735	0.57	5	Yes	Campbell (2016)	Glyma.20G019300	Gm20:1999216‐2021765
*dt1*	Gm19:44778853‐45273019	0.49	53	Yes	Liu et al. (2010).	Glyma.19g194300	Gm19:45183357‐45185175
*Dt2*	Gm18:54576800‐55932579	1.36	37	Yes	Ping et al. (2014)	Glyma.18g273600	Gm18:55638209‐55646547
*S*	Gm13:38244543‐39200524	0.96	27	Yes	Wang et al. (2021).	Glyma.13g287600/Glyma.13g288000	Gm13:38798562‐38839495
*P1*	Gm09:48858116‐49527027	0.67	6	Yes	Liu et al. (2020)	Glyma.09g278000	Gm09:49336440‐49337809
*Pd1*	Gm01:54014967‐54621259	0.61	16	No	Liu et al. (2020)	Glyma.01g240100	Gm01:56405423‐56412746
*Ps*	Gm12:34739995‐35414158	0.67	13	Yes	Liu et al. (2020)	Glyma.12g187200	Gm12:34824285‐34825948
*Td*	Gm03:44372843‐45404915	1.03	5	Yes	Yan et al. (2020)	Glyma.03g258700	Gm03:45300865‐45302850

Abbreviations: bp, base pairs; chr, chromosome, NILs, near‐isogenic lines.

^a^ Number of NILs used for mapping each trait.

^b^ Whether the NIL introgression mapping interval overlapped with the previously published gene model location.

**TABLE 3:** Trait genes mapped using the USDA Soybean Isoline Collection that have been mapped in previous studies, but not yet cloned. The mapped interval coordinates are in reference to the Wm82.a2.v1 genome assembly.

	Results from Isoline Collection mapping				Mapped positions as reported in previous studies		
Trait	Interval (chr:bp)	Interval Size (Mb)	Number of NILs^a^	Overlap^b^	Study	Interval (chr:bp)	Interval size (Mb)
*l2*	Gm03:212961‐568468	0.36	11	Yes	Weiss (1970)	Gm03:0‐467655	0.47
*Lf1*	Gm08:37497004‐39996936	2.50	6	No	Jeong et al. (2017)	Gm08:41292087‐42635713	1.34
*pa1*	Gm12:37092076‐37655070	0.56	12	No^c^	Lee et al. (1999)	Gm11:6971135‐8151771	1.18
*pa2*	Gm13:32301951‐32616365	0.31	12	No	Lee et al. (1999)	Gm13:32866056‐33555523	0.69
*rmd*	Gm16:37159204‐37879369	0.72	7	Yes	Jiang et al. (2019)	Gm16:37102014‐37290074	0.19
*Rsv2 (Rsv1‐r)*	Gm13:30900587‐32225680	1.33	4	Yes	Moon et al. (2009)	Gm13:28912864‐31802676	2.89
*rxp*	Gm17:6577190‐7099140	0.52	28	Yes	Kim et al. (2010)	Gm17:7005472‐7039157	0.03

Abbreviations: bp, base pairs; chr, chromosome, NILs, near‐isogenic lines.

^a^ Number of NILs used for mapping each trait.

^b^ Whether the NIL introgression mapping interval overlapped with the previously published locus location.

^c^ *pa1* is most likely located on Gm12 as reported here and by Bandillo et al. (2017), not on Gm13 as reported by Lee et al. (1999).

**TABLE 4:** Newly mapped traits using the USDA Soybean Isoline Collection. The mapped interval positions listed in this table are the most likely positions. Other potential positions are displayed in the Manhattan plots shown in the Supporting Information 2. Interval size indicates the approximate region containing the trait in megabases (Mb). Number of near‐isogenic lines (NILs) indicates how many NILs were used in the mapping. Recurrent parents indicate which genotypes were used to generate the NILs.

Trait	Interval	Interval size (Mb)	Number of NILs	Recurrent parents
*ab*	Gm13:30677521‐32677565^a^	2.00	3	Clark, Harosoy
*im*	Gm09:11217463‐21494387	10.28	8	Clark, Williams
*lo*	Gm19:49921060‐50681263	0.76	3	Clark, Harosoy
*Np*	Gm10:1336895‐1542603	0.21	6	Clark, Harosoy
*pc*	Gm13:25040804‐26661006	1.62	8	Clark, Harosoy
*Rpm*	Gm15:40841088‐43757793	2.92	18	Clark, Williams, Other

^a^ Base pair positions are in reference to the Wm82.a2.v1 genome assembly.

For the 20 genes that have been previously cloned, 16 had genomic intervals mapped in this study that overlapped the published gene location (Table 2). Relative to the other four genes, genomic intervals were located on the correct chromosome, but the four map intervals fell short (by about 0.22 to 1.78 Mb) of overlapping the published gene location. In two cases (i.e., *G* and *w4*), one NIL had recurrent parent genome segment in the gene region, which resulted in the mapped interval being located either upstream or downstream from the gene location. The reason for this is not clear, though it could be due to an incorrect DNA sample for that NIL. For the *L1* gene, small donor introgressions detected with the SoySNP50K BeadChip were inconsistent in terms of map position. This outcome could possibly have been caused by faulty mapping of the relative positions of the SNPs in this region. In the case of *Pd1*, lack of SNP coverage at the end of Gm01 where this gene was cloned precluded an accurate introgression map.

Seven loci mapped in previous studies, but not yet cloned, were also mapped in this study. For four of these loci, the previously mapped interval overlapped with the intervals reported herein (Table 3). The *Lf1* gene, conferring five‐foliate leaves, was mapped to a 2.5 Mb region on chromosome Gm08, which was 1.3 Mb distant from the interval mapped by Jeong et al. (2017). The introgression mapping conducted in this study also confirmed two previously reported discrepancies in the soybean genetics literature. The *pa1* gene was mapped to Gm13 by Lee et al. (1999), but it was mapped to Gm12 in this study. As pointed out by Bandillo et al. (2017), the original mapping location was likely incorrect due to parology between Gm12 and Gm13 (Lee et al., 2001), causing markers in the region to incorrectly map to the wrong linkage group. Using genome‐wide association mapping, Bandillo et al. (2017) also placed *pa1* on Gm12. Another example was *Rsv1* and *Rsv2*. As reported in previous studies (Chen et al., 2001; Gunduz et al., 2001), *Rsv2* is not a separate locus, but is instead allelic to *Rsv1*. We also confirmed this by mapping what is labeled as *Rsv2* in the Isoline Collection to the known *Rsv1* location. We were able to narrow this interval from a previously reported 2.89 Mb (Moon et al., 2009) to 1.33 Mb.

### 3.3 Newly mapped loci

**FIGURE 3:**
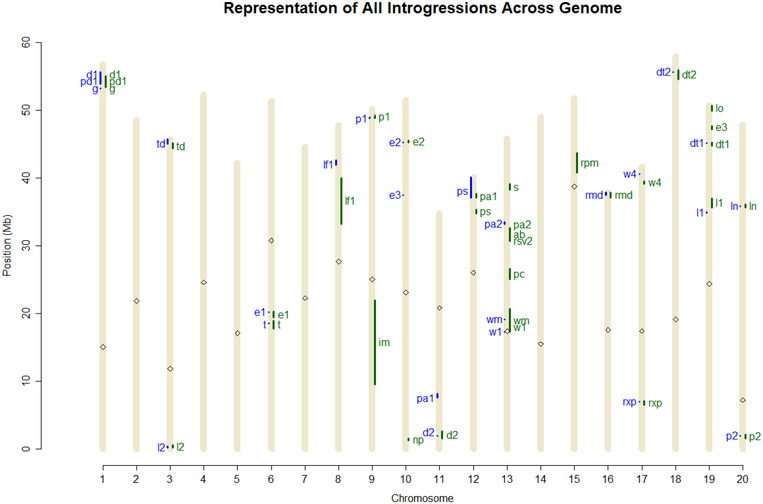
The locations of trait introgressions mapped in this study. Chromosomes are shown as tan lines with centromeres represented by a diamond. Blue indicates a previously mapped locus, whereas green indicates mapping results from this study.

This study mapped genes controlling six traits that had not previously been mapped (Table 4; Figure 3). The length of the genetic intervals mapped ranged from 0.21 Mb (*Np*) to greater than 10 Mb (*im*), with a mean length of 2.76 Mb and a median length of 1.62 Mb. For *lo*, a region on chromosome Gm19 was found to be associated with the trait, but two SNPs on Gm03 were also significant (Supporting Information 3). One of these SNPs on Gm03 SNPs was ruled out because an *lo* NIL carried the RP allele at this SNP, and the other was ruled out because it a was single SNP, which would be a less likely introgression due to linkage drag. The region on Gm19 was favored because it consisted of 44 SNPs spanning a region of 0.76 Mb that did not contain any homozygous RP SNP alleles among NILs (Supporting Information 4). Similar logic was used to select the regions for *pc* and *Rpm* despite multiple significant trait‐SNP introgressions on multiple chromosomes. *Np* showed associations with three genomic regions and *im* showed associations with two genomic regions. In each case the strength of association was much stronger for one region compared to the others (Supporting Information 3). Only one region was associated with *ab*.

Candidate genes within each interval were not analyzed for their likelihood of being causal because each interval contained at least 12 genes, and lack of a full biochemical understanding of each of these traits prevented us from making definitive nominations. The interval sizes, which are informative for future mapping work but preclude detailed candidate gene characterization, were expected based on the relatively small number of NILs sampled and limited number backcross generations used.

## 4 DISCUSSION

### 4.1 Characterization of the USDA Soybean Isoline Collection as a resource for soybean research

NILs are a powerful resource for mapping genes and elucidating their effects on multiple phenotypes (Muehlbauer et al., 1988). Only a few NIL collections exist in crop species that capture multiple traits. Examples include the Bowman NIL collection of barley that includes over 800 NILs capturing genetic variants for dozens of morphological and disease resistance traits (Druka et al., 2011). Another known example is a tomato NIL collection reported by Smith and Ritchie (1983), which includes genes for 170 traits backcrossed into common genetic backgrounds. This paucity of NIL resources makes any such collection a unique and valuable resource for researchers.

The USDA Soybean Isoline Collection—consisting of 611 NILs, more than 100 alleles, and 81 different traits—has been a tremendously valuable resource for the soybean community (Nelson, 2011). The combination of currently available genome‐wide markers along with published phenotype classifications and pedigree information provided an opportunity to perform introgression mapping for traits not yet placed on any map, as well as a means for narrowing the intervals of traits previously mapped to large intervals. We also took the opportunity to genetically characterize the NILs by using publicly available SoySNP50K (Song et al., 2015) to estimate isogenecity and map on‐target and off‐target introgressions.

We distilled the original collection of 611 NILs down to 368 NILs based on the three criteria described in the Methods section, namely backcross derivation; available genotypic data on DP, RP, and NIL; and at least 80% marker identity between NIL and its RP. Four‐hundred forty‐four NILs remained after the first two filtering criteria were applied. Among those 444 NILs, we found that 72 (16%) had a marker similarity to the recurrent parent less than 80%, and these were removed prior to the downstream analyses. The majority of these NILs were BC5‐derived, though some were not, because of their complex pedigrees (Bernard et al., 1991). Our analysis revealed that 18% of the NILs were not isogenic to the recurrent parent, and thus indiscriminately selecting NILs for experiments may be problematic through the introduction of confounding genetic background effects. After discarding the NILs that did not meet the 80% marker identity criteria, the final set of 368 (Table S1) had a mean marker similarity to the recurrent parent of 95.7%, with the median being 96.8%. While this is still slightly less than the expectation for a BC5‐derived NIL (98.4%), this subset represented a high‐quality set of NILs on which we performed the mapping analysis. The information provided here will help inform researchers on the selection of NILs for their studies.

An analysis of the marker‐delineated introgression segments that were present in the 368 NILs used for mapping revealed a wide range of introgression interval lengths. Some off‐target introgressions (unlinked to the intended QTL) were as small as 2.6 kb, while others were as large of 54.8 Mb, which is roughly equivalent to one entire chromosome of the soybean genome. The mean off‐target introgression interval length was 6.4 Mb, representing 0.6% of the total genome size, or 11.6% of the average soybean chromosome size. For comparison, Druka et al. (2011) found that a set of 426 Bowman barley NILs had off‐target introgressed segments less than 50 cM, representing <3% of the genetic map.

### 4.2 Trait mapping

Introgression mapping based on a comparative marker analysis of trait‐specific NIL/RP/DP sets is a powerful technique that can assist in mapping large‐effect single genes controlling qualitative traits, with numerous such examples in soybean (e.g., Cairo et al., 2002; Mian et al., 1999; Tasma & Shoemaker, 2003). The USDA Soybean Isoline Collection is a unique resource that contains many dozens of different alleles, allowing for the possibility of simultaneously mapping multiple traits. Muehlbauer et al. (1991) attempted this in the early days of genetic mapping. These authors used 15 RFLP loci to make four marker‐trait associations: *p_1_*, *r*, *Lf_1,_* and *ab*. Moreover, Muehlbauer et al. (1991), despite being constrained by the marker technology and resources of the time, was able to place loci on tentative linkage groups. In the current study, we were able to map 33 nuclear genes controlling qualitative traits in this collection. Twenty‐seven of these had been previously mapped. We mapped 21 to the same published mapped intervals. Although our mapped positions for six of the 21 genes did not overlap with published map intervals, prior mapping intervals and our intervals were always adjacent to each other, even if separated by gaps ranging from 200 kb to nearly 6 Mb.

An interesting example includes the case of *Rsv2*, which we showed, as expected, maps to the *Rsv1* locus (Table 3). After the discovery of *Im*, another gene conferring resistance to SMV (Soybean mosaic virus; *Potyvirus*) was discovered and named *Rsv* (later re‐named as *Rsv1*; Kiihl & Hartwig, 1979). The existence of a separate locus controlling SMV resistance was claimed by Buzzell and Tu (1984) after the analysis of a cross between 'OX670' (a line resistant to SMV, but resistance source unknown) and an 'L78‐379', a line shown to have *Rsv1*. The resulting F_2_ population was shown to segregate, suggesting that OX670 possesses a unique source of SMV resistance, which the authors named *Rsv2*. As the cultivar Raiden was the only ancestor of OX670 known to be resistant to SMV, Buzzell and Tu (1984) assumed the resistance was inherited from Raiden. However, Chen et al. (2001) clearly showed that the resistance gene in Raiden is allelic to *Rsv1*, suggesting that OX670 either did not inherit its resistance from Raiden, or it has multiple genes inherited from different ancestors conferring resistance. Indeed, further studies showed that OX670 possesses a resistance allele, *Rsv1‐r*, inherited from Raiden in addition to a *Rsv3* allele contributed by the other parent of OX670, which was Harosoy*. Rsv3* was reported by Buzzell and Tu (1989) subsequent to the publication of Buzzell and Tu (1984) on *Rsv2*. Therefore, there is no evidence that the separate locus named *Rsv2* actually exists. Bernard et al. (1991) listed Raiden as the donor parent for the supposed *Rsv2* gene, but it is clear now from this previous work that Raiden contributed *Rsv1‐r*, not the non‐existent *Rsv2*.

Aside from the foregoing complications, we were able to map six genes not previously placed on any map. These loci include *ab* (delayed leaf abscission), *im* (non‐mottled seed coats in plants exhibiting SMV infection), *lo* (oval leaf), *pc* (curly pubescence), *Rpm* (resistance to downy mildew), and *Np* (tolerance to low phosphorus). The following paragraphs briefly review the importance and history of each trait.

### 4.3 *ab* (delayed leaf abscission)

Leaf abscission occurs with the development of a layer of cells at the petiole base called the abscission layer. As the plant matures and begins to senesce, auxin levels decline and the abscission layer weakens and separates, causing the plants to drop their leaves at maturity (Hopkins, 1999). Some soybean varieties do not drop their leaves at maturity, a characteristic known as delayed abscission. This was a desirable characteristic of some varieties when soybeans were grown for forage (Probst, 1950), but when soybeans began to be grown for seed, delayed abscission complicated harvesting and was generally deemed undesirable. In analyzing progenies from six crosses between parents showing normal abscission and delayed abscission, Probst (1950) showed that the delayed abscission phenotype observed in the cultivar Kingwa was controlled by a single gene. The abscised phenotype, conferred by the *Ab* allele, displayed dominance whereas the delayed abscission phenotype (*ab*) displayed recessiveness (Probst, 1950). Remarkably few studies on this trait have been performed, and no mapping results have been reported. Muehlbauer et al. (1991) mapped *ab* to linkage group F (which corresponds to Gm13) (Soybase.org), albeit to the chromosome level rather than interval level. We were able to map *ab* to a 2 Mb interval on the long arm of Gm13 (Figure 3; Table 2) where it is tightly linked to *pa2* and *rsv2* (Figure 3). Future studies aimed at fine mapping and cloning *ab* could contribute knowledge on the genetic control of leaf abscission in soybean and possibly open avenues in developing molecular techniques towards altering this trait.

### 4.4 *Im* (resistance to soybean mosaic virus)

SMV can cause plant stunting, reduced yield, and mottled seed coats (Wilcox, 1983). An early source of resistance to SMV was identified in the cultivar Merit by Cooper (1966), who determined that SMV resistance was controlled by a single gene, designated *Im*, with complete to partial dominance. It was later shown that *Im* did not confer complete immunity to SMV, but instead just limited seed coat mottling and transmission of the virus to the mature seed (Wilcox, 1983). Unfortunately, new strains of SMV have proliferated that have overcome many genes for host plant resistance, including *Im*, such that it now provides virtually no resistance to SMV. In fact, Bernard et al. (1991) noted that *Im* genotypes showed mottling in years just prior to the report. Thus, between 1972 and 1991, the *Im* isolines lost the ability to resist seed coat mottling under SMV pressure, even with selection. We mapped *Im* to a large interval (Table 4) on Gm09 for the first time, although another statistically significant peak does exist on Gm18 which cannot be ruled out (Supporting Information 4). The development of many SMV strains that easily overcome *Im* is the likely reason why no subsequent genetic studies on this gene have been performed in the era of molecular mapping. Still, identifying the causal gene underlying *Im* could contribute to an enhanced understanding of virus resistance in soybean which ultimately may lead to enhanced genetic resistance through transgenesis or gene editing.

### 4.5 *lo* (oval leaflet)

Most soybeans leaflets are ovate in shape, but variations exist. For example, the *ln* allele on Gm20 produces narrow, lanceolate‐type leaflets (Jeong et al., 2012). The *ln* allele also has a pleiotropic effect on seed size because *lnln* genotypes with lanceolate leaves also tended to have pods with four smaller seeds compared to *LnLn* genotypes with normal ovate leaflets that do not produce such 4‐seed pods (Jeong et al., 2012). Another gene known as *lo* also has an effect on leaflet shape (Weiss, 1970). This locus was first discovered by Domingo (1945), who determined the inheritance of oval leaflets to be controlled by a single gene with the *Lo* allele conferring normal leaflets that are dominant to oval. He also noted that *lolo* genotypes not only exhibited oval leaflets, but also tended to have a high frequency of 2‐seed (vis‐a‐vis fewer 3‐seed) pods that resulted in fewer and larger seeds (Domingo, 1945). To our knowledge, no additional studies on the genetics of oval leaflets in soybean have been performed. We mapped, for the first time, *lo* to a region on the end of Gm19 spanning 0.76 Mb. Two SNPs on Gm03 did reach statistical significance and so Gm03 cannot be ruled out, but the number of SNPs showing an introduction on Gm19 is more consistent with an introgression and thus is the more likely position (Supporting Information 4). This initial mapping will be useful for those interested in initiating a map‐based cloning of *lo* that would help reveal is physiological function.

### 4.6 *Np* (tolerance to phosphorus)

Howell (1954) found marked differences between soybean cultivars in their tolerance to high levels of phosphorus, with 'Chief' being tolerant and 'Lincoln' and 'Illini' being more sensitive. Bernard and Howell (1964) investigated the genetic basis of the tolerance found in Chief by crossing it to Clark, another sensitive cultivar, and then deriving BC3 lines using Clark as the RP. Subsequent inheritance studies using Clark and the BC3‐tolerant lines indicated a major gene controlling phosphorus tolerance displaying Mendelian inheritance. Bernard and Howell (1964) symbolized the allele conferring phosphorus tolerance as *Np*, and the allele conferring phosphorus sensitivity as *np*. The NILs in the Isoline Collection were made using Chief as the donor parent and Clark and Harosoy as the recurrent parents. Raboy (1997) reported that tolerance to the phosphorus toxicity exhibited by *NpNp* genotypes was related to the lack up phosphorus uptake at the root‐rhizosphere interface. It was found that phytic acid in the seed of *npnp* soybean lines increased linearly with increasing phosphorus levels in the soil. *NpNp* genotypes showed similar levels of phytic acid as *npnp* genotypes when grown in low and moderate phosphorus nutrient levels, but *NpNp* genotypes accumulated less seed phytic acid than *npnp* genotypes when grown in high phosphorus levels. However, non‐phytic levels of phosphorus were not affected (Raboy, 1997). To our knowledge, no genetic mapping studies have been conducted for *Np*. Our introgression mapping analysis placed *Np* on chromosome 10 within a 0.21 Mb interval, although on SNP one Gm01 and two SNPs on Gm02 reached significance and thus these positions cannot be ruled out as possible loci corresponding to *Np* (Supporting Information 4). Future genetic studies to identify the causal polymorphism underlying *Np* could potentially illuminate mechanisms for nutrient uptake and possibly transport, ultimately helping to identify genetic solutions to nutrient deficiency and toxicity.

### 4.7 *pc* (curly pubescence)

Soybean plants are typically covered by a dense layer of trichomes, known as pubescence. Almost all cultivars have a normal pubescence, but variants differing from this wild‐type exist, and have been found to be largely controlled by single genes (Bernard & Singh, 1969). Early soybean researchers reported on a type of pubescence found in Japanese varieties that was characterized as flat and curly (Takahashi & Fukuyama, 1919). Bernard and Singh (1969) derived a progeny line (named 'T141') from the Japanese cultivar 'Oni Hadaka' (PI 84987) and crossed it to Clark and Harosoy to derive a F2 population. It was determined that the curly pubescence phenotype was controlled by a single recessive allele symbolized by *pc*. It has been noted that curly pubescence makes plants more susceptible to leaf hopper damage (Bernard & Singh, 1969; Specht et al., 1985). A 1.6 Mb interval on Gm13 is the most likely introgression corresponding to *pc* because of its consistency among NILs and size, but significant SNPs on Gm01, Gm02, and Gm20 cannot be ruled out (Supporting Information 4).

### 4.8 *Rpm* (resistance to downy mildew)

Downy mildew is a widespread disease that, fortunately, does not result in large yield losses in soybean, but it can reduce seed size and quality (Taguchi‐Shiobara et al., 2019; Wilcox, 1983). This disease can thus have an impact on food‐type soybeans. Bernard and Cremeens (1971) determined that the resistance in the cultivar Kanrich was controlled by a single gene pair with *Rpm* symbolizing the allele exhibiting partial dominance for resistance over the *rpm* allele for susceptibility. Our analysis mapped this gene for the first time to the short arm of Gm15 within a 2.92 Mb interval, although there is also evidence for its presence on Gm03 (Supporting Information 4). Chowdhury et al. (2002) identified Random Amplified Polymorphic DNA markers that were linked to resistance to downy mildew for the purpose of marker‐assisted selection in soybean, but the chromosomal location of the resistance locus was not ascertained.

## 5 CONCLUSION

By combining genome‐wide marker data collected on the USDA Soybean Isoline Collection along with phenotype and pedigree data, we were able to genetically characterize the collection, thereby narrowing the mapping intervals for three genes (*l2*, *pa1*, *Rsv1*), and identifying, for the first time, likely map positions of six other genes (*ab*, *Im*, *lo*, *Np*, *pc*, *Rpm*). The NIL introgression mapping technique generated map position intervals that, in most instances, spanned known genomic positions of previously mapped (or cloned) genes. Though there were a few cases in which the map position interval did not span a known gene position, the physical distance between the latter and nearest flanking side of interval was small. Though this suggests that the technique is reasonably reliable for use as a first‐step when mapping heretofore unmapped genes that are present in NILs, one should nevertheless conduct follow‐up confirmatory mapping if the ultimate goal is a map‐based cloning effort.

## AUTHOR CONTRIBUTIONS

**Erin Gilbert**: Data curation; formal analysis; methodology; software; visualization; writing–original draft. **Ryan Merry**: Data curation; formal analysis; writing–review and editing. **Benjamin W. Campbell**: Conceptualization; data curation; writing–review and editing. **Robert M. Stupar**: Conceptualization; writing–review and editing. **Aaron J. Lorenz**: Conceptualization; supervision; writing–original draft.

## CONFLICT OF INTEREST STATEMENT

The authors declare no conflict of interest.

## Supporting information

Supplementary File 1

Supplementary File 2

Supplementary File 3

Supplementary File 4

Table S1 Recurrent parent (RP) marker similarity of each NIL.
